# PhenoSpace: A Shiny application to visualize trait data in the phenotypic space of the global spectrum of plant form and function

**DOI:** 10.1002/ece3.6928

**Published:** 2021-01-26

**Authors:** Jules Segrestin, Kevin Sartori, Marie‐Laure Navas, Jens Kattge, Sandra Díaz, Eric Garnier

**Affiliations:** ^1^ CEFE, Univ Montpellier, CNRS EPHE, IRD Univ Paul Valery Montpellier 3 Montpellier France; ^2^ CEFE, Univ Montpellier, CNRS EPHE, IRD, Institut Agro Univ Paul Valéry Montpellier 3 Montpellier France; ^3^ Max Planck Institute for Biogeochemistry Jena Germany; ^4^ German Centre for Integrative Biodiversity Research (iDiv) Halle‐Jena‐Leipzig Leipzig Germany; ^5^ Instituto Multidisciplinario de Biología Vegetal (IMBIV) CONICET and FCEFyN Universidad Nacional de Córdoba Córdoba Argentina

**Keywords:** comparative plant ecology, functional dimensions, plant phenotypes, plant traits, trait variation, user‐friendly application

## Abstract

1. A recent analysis of variation in six major traits conducted on a large worldwide sample of vascular plant species showed that three‐quarters of trait variation was captured by a two‐dimensional global spectrum of plant form and function (“global spectrum” hereafter). We developed the PhenoSpace application, whose aim is to visualize and export the position of any individual/population/species in the phenotypic space of the global spectrum.
2. PhenoSpace is a Shiny application that helps users to manipulate and visualize data pertaining to the global spectrum of plant form and function. It is freely accessible at the following URL: https://shiny.cefe.cnrs.fr/PhenoSpace/.
3. PhenoSpace has three main functionalities. First, it allows users to visualize the phenotypic space of the global spectrum using different combinations of traits and growth forms. Second, trait data from any new user‐defined dataset can be projected onto the phenotypic space of the global spectrum, provided that at least two of the six traits are available. Finally, figures produced and loadings of the imported data on the PCA axes can be downloaded, allowing users to conduct further analyses.
4. PhenoSpace fulfills the practical goal of positioning plants in the phenotypic space of the global spectrum, making it possible to compare trait variation at any level of organization against the worldwide background. This serves a major aim of comparative plant ecology, which is to put specific sets of individuals, populations or species into a broader context, facilitating comparison and synthesis of results across different continents and environments using relevant indicators of plant design and function.

## Graphical abstract

PhenoSpace is a shiny application that fulfills the practical goal of positioning plants in the phenotypic space of the global spectrum of plant form and function, making it possible to compare trait variation at any level of organization against the worldwide background.

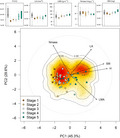

## 1 INTRODUCTION

Traits, which are measurable properties of individuals related to their functioning and modulating their fitness (Calow, 1987; McGill et al., 2006; Violle et al., 2007), are key to study biodiversity from a functional perspective (Enquist et al., 2015; Garnier et al., 2016; Lavorel et al., 2007 for reviews). In plants, an established body of research has been devoted to the identification of key traits allowing a synthetic description of phenotypes in a way that is relevant to their functioning and ecology (Grime, 1977; Laughlin, 2014; Weiher et al., 1999; Westoby, 1998). A recent study conducted on the largest dataset ever compiled of six major traits critical to growth, survival, and reproduction and measured on a sample of vascular plant species distributed worldwide, showed that three‐quarters of trait variation could be captured in a two‐dimensional “global spectrum of plant form and function” (“global spectrum” hereafter: Díaz et al., 2016). One major dimension reflects the size of whole plants and their parts, while the other represents the leaf economics spectrum (Wright et al., 2004), which runs from quick to slow return on investments of nutrients or dry mass in leaves. As a major advance in our understanding of plant phenotypes, this global spectrum provides a backdrop for describing species from a functional perspective using relevant axes of variation (discussed in Díaz et al., 2016; Westoby et al., 2002). We, therefore, expect extensive use of this phenotypic space as a reference in plant trait‐based research (907 citations to the Díaz et al., 2016 paper in Google Scholar as of 4 August 2020). In this context, we developed the application PhenoSpace, which fulfills the practical goal of positioning plants in the phenotypic space of the global spectrum, making it possible to compare trait variation at any level of organization against the worldwide background. This serves a major aim of comparative plant ecology, which is to put specific sets of individuals, populations or species into a broader context, facilitating comparison and synthesis of results across different continents and environments using relevant functional axes. More precisely, PhenoSpace allows one to: (a) visualize the outcome of various multivariate analyses run with the original dataset compiled by Díaz et al. (2016) or subsets thereof; (b) project and visualize any user‐defined dataset in the phenotypic space of the global spectrum; and (c) download figures produced by the user and/or coordinates of the imported dataset on the PC axes of plant size and the leaf economics spectrum.

## 2 THE FUNCTIONALITIES OF PHENOSPACE

Shiny is an *R* package that makes it easy to build interactive web applications from *R* (Chang et al., 2019). PhenoSpace, the Shiny application presented here, is designed to help users manipulate and visualize data pertaining to the global spectrum. It is available at the following URL: https://shiny.cefe.cnrs.fr/PhenoSpace/. Its functionalities, accessible from three different tabs, are described below.

### 2.1 Visualizing the phenotypic space of the global spectrum

The first functionality of PhenoSpace (“Customize the PCA” tab) is to display the outcomes of multivariate analyses based on the original dataset explored by Díaz et al. (2016).

Six traits are involved in the definition of the phenotypic space of the global spectrum (Table 1 for definitions, units of expression, and functional significance): adult plant height (H), stem specific density (SSD), the area of a leaf (LA), leaf mass *per* area (LMA), leaf nitrogen content *per* unit mass (N_mass_), and seed or spore dry mass (SM). The species geometric mean values of the six traits were log‐transformed to fulfill normality assumptions, centered and scaled before the analysis. PhenoSpace allows dynamic visualization of the loadings of the 2,214 species available in the original dataset on any combination of components of the principal components analyses (PCA) run either on these six traits or on any user‐defined subsets of these traits. The user can also choose to display data for species of all growth forms available in the original dataset (herbs, shrubs, trees, and others), or only for a selection of these growth forms. When a selection is made, a new PCA is run using the corresponding subset of the dataset.

**Table 1:** Traits involved in the phenotypic space of the global spectrum of plant form and function (Díaz et al., 2016) and their functional significance (Garnier et al., 2016; Pérez‐Harguindeguy et al., 2013 for details), together with their abbreviations and units. Definitions are taken from the thesaurus of plant characteristics (TOP: Garnier et al., 2017)

Trait	Definition	Functional significance	Abbreviation	Unit	Range of variation in the Díaz et al. (2016) dataset
Adult plant height	The shortest distance between the upper boundary of the photosynthetic tissues of an adult plant and the ground level	Light capture, above‐ground competition, dispersal distance of seeds	H	m	0.001–90
Stem specific density	The ratio of the mass of the stem or a unit thereof assessed after drying to its volume assessed without drying	Growth potential versus mortality risk from biomechanical or hydraulic failure	SSD	mg/mm^3^	0.06–1.39
Leaf area	The area of a leaf in the one‐sided projection	Light interception, energy balance	LA	mm^2^	0.79–2.79 × 10^6^
Leaf mass per area	The ratio of the dry mass of a leaf to its area	Photosynthetic rate, leaf longevity, seedling relative growth rate	LMA	g/m^2^	4.9–1,507
Leaf nitrogen content per unit mass	The ratio of the quantity of nitrogen in the leaf and its dry mass	Protein content (RubisCo), photosynthetic rate	N_mass_	mg/g	2.48–68.98
Seed dry mass	The mass of a seed assessed after drying	Dispersal and regeneration strategy, seedling competition	SM	mg	3.0 × 10^−4^ to 2.05 × 10^a^

^a^ Excluding Pteridophytes.

**Figure 1:**
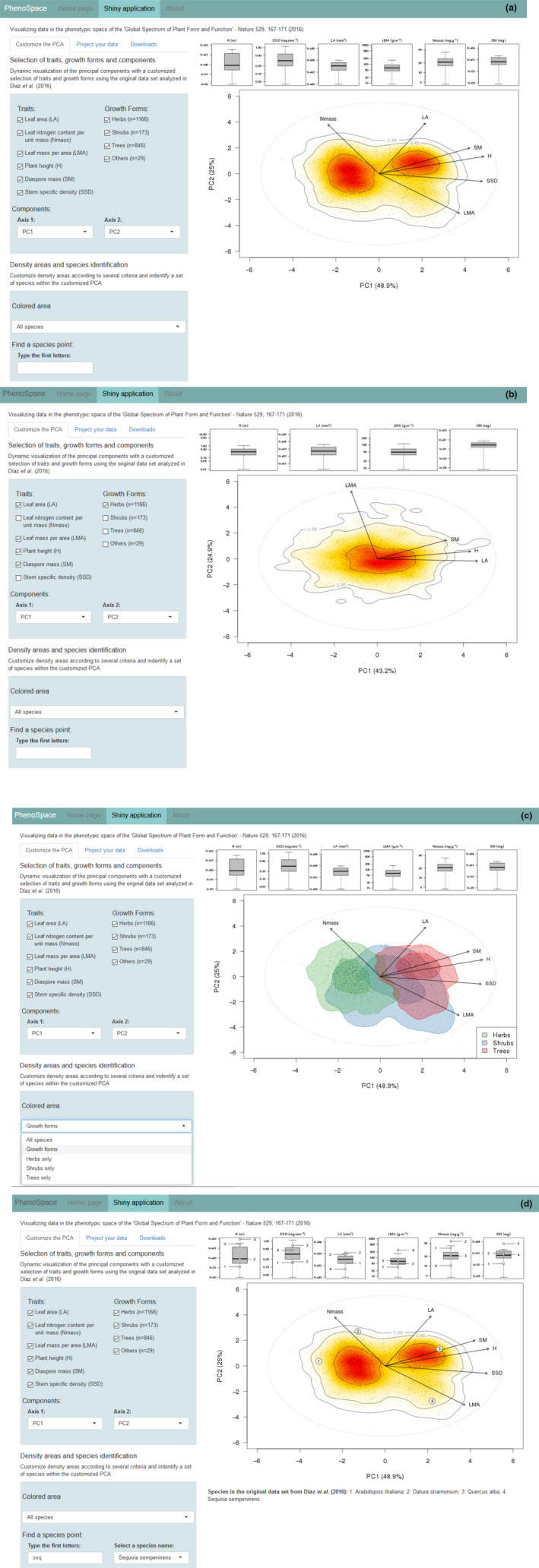
Screenshots showing the PhenoSpace page displays (“Customize the PCA” tab) for different selections of variables and species groups. (a) all traits and species from the original dataset analyzed in Díaz et al. (2016); (b) plots for herbaceous species using only four traits: Plant height (H), Leaf area (LA), Leaf mass *per* area (LMA) and Seed dry mass (SM); (c) distinct density areas for the three main growth forms (for each growth form, the 0.50 and 0.95 density areas are represented); (d) positions of four species (1 *Arabidopsis thaliana*, 2 *Datura stramonium*, 3 *Quercus alba,* and 4 *Sequoia giganteum*) in the plane defined by the first two components of the PCA run on the whole dataset

**Figure 2:**
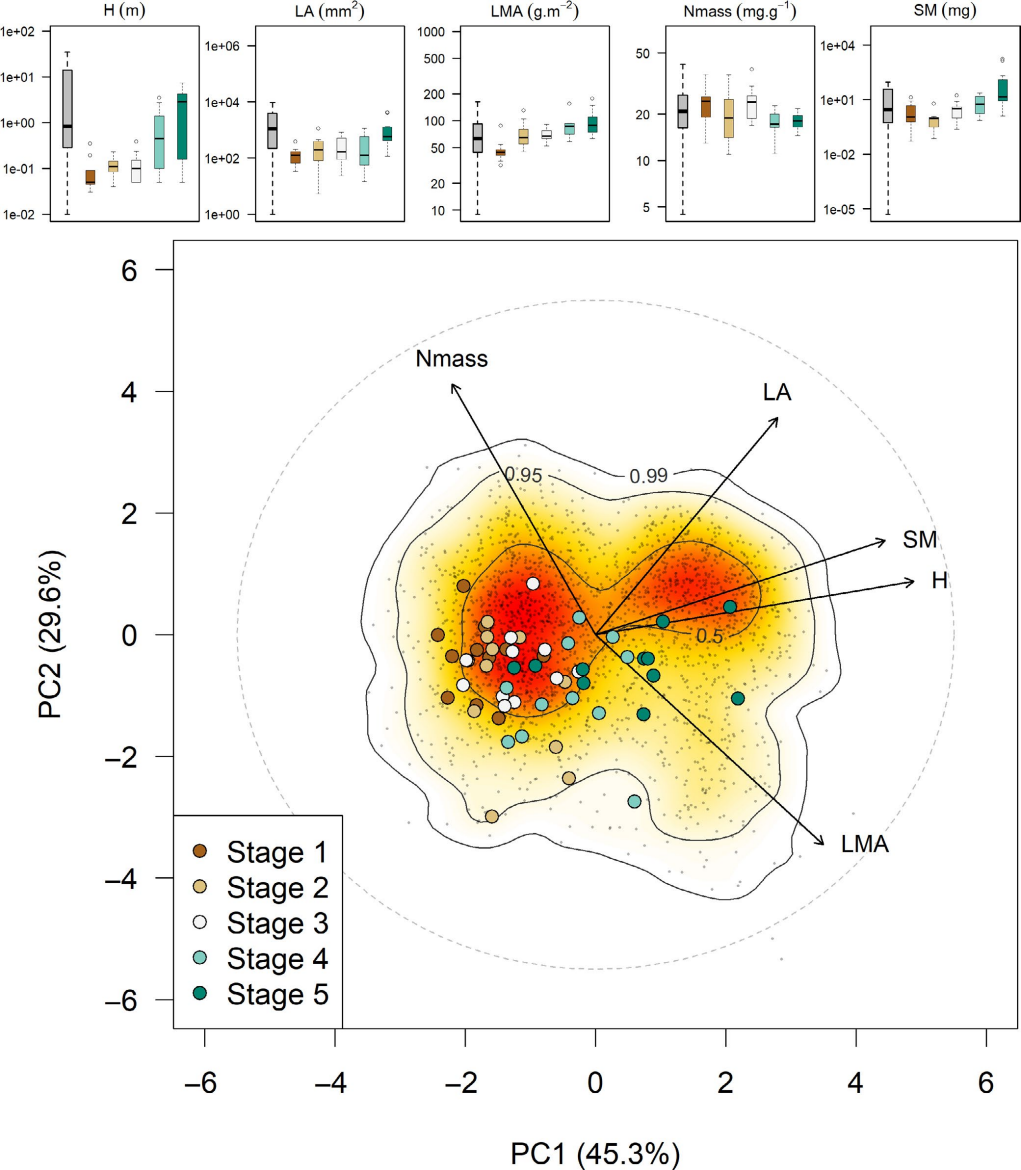
Downloaded figure from PhenoSpace showing the projection of 55 species representative of five successional (from stage 1: recently abandoned agricultural fields or rangelands to stage 5: mature forests) stages in Mediterranean southern France (see Navas et al., 2010) on the global spectrum. Specific stem density was missing in the uploaded dataset and was therefore excluded from the analysis. The boxplots in the upper part show the trait distributions in the global spectrum and in each successional stage separately. See Table 1 for trait abbreviations

Boxplots at the top of the page show the ranges of values for the selected traits covered by the selected species (Figure 1). By default, principal components 1 and 2 of the PCA run on all six traits for all species are displayed (Figure 1a) which corresponds to Figure 2a shown in Díaz et al. (2016). The correlation circle (showing the correlation between the traits and the principal components) was superimposed to the plot using a scaling factor. Arrows ending close to the dotted circle describe traits that are well represented on the selected plane. Figure 1b shows the result of a new PCA in which only four traits (H, LA, LMA, and SM) and one growth form (herbaceous species) are used.

On the PCA representation, a density area showing the occurrence probability of species in the trait space is shown as a color gradient (from red = highest to white = lowest) as in Díaz et al. (2016). In PhenoSpace, the “Colored area” section allows representing alternative density areas using subsets of species according to their growth forms (Figure 1c for example).

Finally, the position of a set of species selected by the user among the 2,214 species available in the original dataset can be explicitly represented in the dynamically displayed phenotypic space (Figure 1d for examples).

### 2.2 Plotting data onto the phenotypic space of the global spectrum

The second functionality of PhenoSpace (“Project your data” tab) allows the projection of new data from an uploaded file onto the user‐defined phenotypic space of the global spectrum. Therefore, any dataset providing values for at least two of the six traits can be easily compared with the worldwide trait distribution.

The first step is to prepare a csv file that contains the data to be projected. Trait values should be organized in columns whose headings correspond to the abbreviations of trait names spelt out exactly as shown in Table 1. Trait values should be expressed in the units shown in Table 1, and no data transformation is required before the upload. Each line of the dataset can describe an individual, a population, a species or even a community (“entity” hereafter). Entities with missing values (coded NA) are excluded from the analysis. No data completion or gap‐filling method is performed in the application. If it is considered useful for a specific dataset, it should be performed by the user before uploading the file. Extracolumns are allowed and can subsequently be used to identify subsets of data on the plot (see below).

The following conditions must be verified on the application setting before uploading the file: (a) the separator and decimal characters are well specified on the “Project your data” tab; and (b) if the imported file misses any of the six traits from the original dataset, they should be unticked on the “Customize the PCA” tab. The csv file can then be uploaded by clicking on the “Browse button” on the “Project your data” tab.

Once the importation is completed, entity trait values from the imported dataset are visualized as (a) boxplots drawn for each trait, displayed next to those showing the distribution of trait values from the original global spectrum dataset (top of the page); and (b) additional data points appearing on the selected plane of the PCA (Figure 2). For the latter, the imported data are treated as inactive individuals in the PCA: The projection of each entity on the PCA planes is calculated using the trait loadings on the principal components of the global spectrum (see section 2.1). The background plane of the PCA onto which the data are projected is thus unaltered.

The optional extracolumns in the data file can be used to identify data points according to the attributes of the criteria specified in the column (which can be quantitative or qualitative). Here, we show an example of the projection of 55 plant species representative of five successional stages (from recently abandoned agricultural fields or rangelands to mature forests) in Mediterranean southern France (see Navas et al., 2010) on the global spectrum (Figure 2). The successional stage was used as qualitative criteria to color the species points in the PhenoSpace app.

### 2.3 Download figures and tables

**Figure 3:**
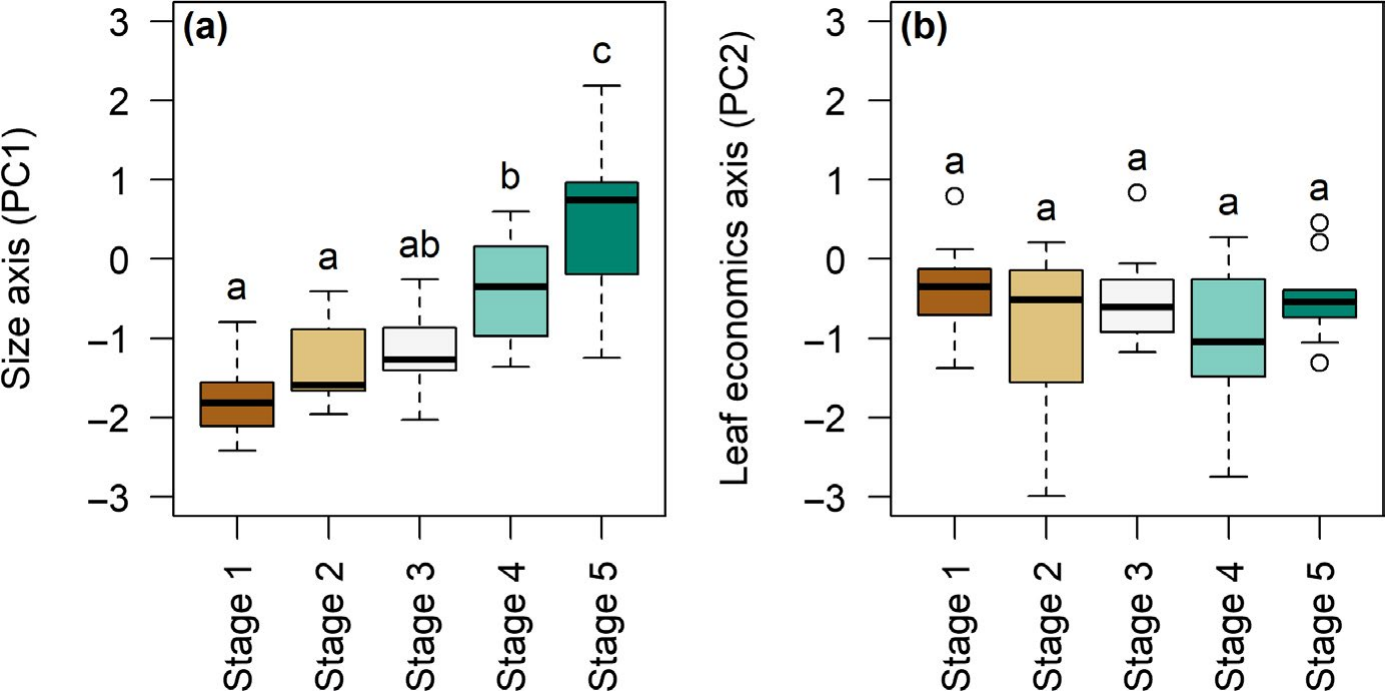
Post hoc analysis using the coordinates of the 55 species from Navas et al. (2010) on the global spectrum axes (projection shown in Figure 2). Boxplots represent the 0.25, 0.5, and 0.75 quantiles (colored boxes) and the range of values (whiskers) covered by species in each successional stage. Boxplots that share the same letter are not significantly different: post hoc Tukey test, *p* value > .05

The third tab (Downloads) allows the user to save the figures in a *png*, *pdf,* or *svg* format specifying the image size and resolution (for *png* files). Color changes and the manipulation of graphic elements can be easily done on vector graphics (*svg* format) once downloaded. When a dataset is projected onto the PCA, entity coordinates on the principal components can be downloaded in an automatically produced csv file. These coordinates are added to the originally uploaded table as extracolumns. The file also contains a header describing the list of traits and the number of species from the original dataset that has been used to run the PCA (see section 2.1). These coordinates can be used for further analyses. For example, the coordinates of the 55 plants species studied in Navas et al. (2010) on the two first components of the global spectrum were downloaded from PhenoSpace. The five successional stages were then compared using a posthoc analysis on these coordinates (Tukey tests, Figure 3). We found that early‐successional species had lower values than late‐successional species on the size axis (PC1) while no difference between stages was found on the leaf economics axis (PC2).

## 3 DISCUSSION AND CONCLUSION

The final sentence of the Díaz et al. (2016) paper reads: “The global spectrum of plant form and function is thus, in a sense, a galactic plane within which we can position any plant ‐from star anise to sunflower ‐ based on its traits.” More specifically, it provides a common basis for further trait‐based multivariate analyses. PhenoSpace helps to fulfill the practical goal of positioning plants quickly and simply in this phenotypic space, making it possible to compare trait variation at any level of organization against the worldwide background. This can be done not only for species, but also for populations or genotypes within species, or even for higher taxonomic ranks or communities. PhenoSpace is a service to the community, which is in principle similar to, *for example,* the taxonomic name resolution service (TNRS, Boyle et al., 2013) or the calculator developed by Pierce et al. (2017) to allocate positions to species/populations in Grime's plant strategy scheme (see below). It provides a web interface that allows one to compute entities coordinates in the global spectrum in a very simple way what could otherwise be done only with much more effort or not at all.

Here, we discuss three benefits of using this phenotypic space as a reference, instead of running independent analyses for new datasets. First, principal component analyses are generally preceded by scaling of trait data aiming to compare the variation of several traits, although expressed in different units and covering various ranges of values. This procedure often uses the distribution observed in the dataset as a reference, giving equal weightings to the range of values covered by each trait in the construction of principal components. However, it is rarely assessed whether the observed distributions cover a significant range of values, as compared to global datasets. The imbalance of trait representativeness can lead to a distortion of the resulting PCA. For example, centering and scaling a dataset that includes low variation in size traits but large variation in leaf traits, regarding global distribution, will result in a PCA, which over‐disperses entities on the size axis as compared to the leaf axis. In PhenoSpace, we used the worldwide distribution of trait values to center and scale the imported trait data, resulting in a more accurate description of the multivariate trait variation. Second, a low range of values or a small number of entities can result in an inaccurate estimation of trait covariations. In these cases, one can consider that the use of large datasets such as the original dataset explored by Díaz et al. (2016) is more appropriate to construct a meaningful multivariate space. PhenoSpace relies on this idea as imported data are considered as inactive entities in the PCA. Finally, there is an increasing interest in the description of volume and shapes of phenotypic hypervolumes (Blonder, 2018). The global spectrum provides a standardized multivariate space for each set of traits, from which hypervolumes of projected data can be computed (using the download functionality, see section 2.3) and easily compared.

PhenoSpace is an open source project (available on a GitHub repository at: https://github.com/jsegrestin/phenospace). The application will be regularly updated to make sure that it provides the most detailed phenotypic space of plants as our understanding and description of plant phenotype improves. We will therefore work in close collaboration with plant trait databases (*e.g.,* the TRY database, see Kattge et al., 2011, 2020) to include new species and/or traits in future versions of the application. Our decision rule will be to include a new trait when data are available for a large amount of species in common with the current global spectrum and displays a wide range of variation. The version number, a report detailing the new features, and links to old versions of the application will be available on the webpage.

PhenoSpace could also be further developed to position entities in phenotypic spaces already defined with sets of traits different from those of the global spectrum. Two schemes would be particularly interesting in this perspective. The first one is that of the leaf economics spectrum (Wright et al., 2004), which is extensively used to position species along an axis of resource acquisition and conservation, and involves the six following leaf traits: photosynthetic and respiration rates, LMA, N_mass_, leaf phosphorus content *per* unit mass and leaf life span. The second one is the CSR ecological strategy scheme (Grime, 1977, 2001), in which entities are assigned scores on axes characterizing their degree of competitive ability (C), stress‐tolerance (S), and ruderality (R) from the values of their traits. Using either the algorithm developed by Hodgson et al. (1999) based on seven traits or that recently proposed by Pierce et al. (2017) based on three leaf traits, any species could then be positioned in the CSR triangular ordination in a way similar to what has been done here to place species in the PCA planes.

The current and future versions of PhenoSpace will serve a major aim of comparative plant ecology, which is to put specific sets of individuals/populations/species/communities into a broader context (Díaz et al., 2016), allowing one to synthesize results across different continents and environments using relevant indicators of plant functioning (cf. Westoby et al., 2002). Future developments may not only be based on existing schemes of phenotypic spaces but could also incorporate other relevant functional dimensions as our understanding of plant phenotypes improves.

## CONFLICT OF INTEREST

The authors state that there is no conflict of interest.

## AUTHOR CONTRIBUTION

**Jules Segrestin:** Conceptualization (lead); Project administration (lead); Software (lead); Writing‐original draft (lead); Writing‐review & editing (lead). **Kevin Sartori:** Conceptualization (lead); Writing‐review & editing (supporting). **Marie‐Laure Navas:** Resources (equal); Writing‐review & editing (supporting). **Jens Kattge:** Data curation (lead); Resources (lead); Writing‐review & editing (equal). **Sandra Díaz:** Data curation (lead); Resources (lead); Writing‐review & editing (equal). **Eric Garnier:** Writing‐original draft (lead); Writing‐review & editing (lead).

## Data Availability

The code of the application is available on a GitHub repository at: https://github.com/jsegrestin/phenospace. All datasets used in this paper are from published studies. To avoid conflicts of interest, the dataset of the global spectrum will be published soon as a datapaper and a link will be available on the webpage once it is published.
